# Carrageenan as an Ecological Alternative of Polyvinylidene Difluoride Binder for Li-S Batteries

**DOI:** 10.3390/ma14195578

**Published:** 2021-09-26

**Authors:** Tomáš Kazda, Dominika Capková, Kamil Jaššo, Andrea Fedorková Straková, Elena Shembel, Alex Markevich, Marie Sedlaříková

**Affiliations:** 1 Department of Electrical and Electronic Technology, Faculty of Electrical Engineering and Communication, Brno University of Technology, Technická 10, 61600 Brno, Czech Republic; xjasso00@stud.feec.vutbr.cz (K.J.); sedlara@feec.vutbr.cz (M.S.); 2 Department of Physical Chemistry, Faculty of Sciences, Pavol Jozef Šafárik University in Košice, Moyzesova 11, 04154 Košice, Slovakia; dominika.capkova@student.upjs.sk (D.C.); andrea.fedorkova@upjs.sk (A.F.S.); 3 Ukrainian State University of Chemical Technology, 49000 Dnipro, Ukraine; eshembel@enerize.com (E.S.); alexmarkchem@gmail.com (A.M.)

**Keywords:** lithium, sulfur, binder, battery, cathode, Li-S battery, carrageenan, pouch cell

## Abstract

Lithium-sulfur batteries are one of the most promising battery systems nowadays. However, this system is still not suitable for practical application because of the number of shortcomings that limit its cycle life. One of the main problems related to this system is the volumetric change during cycling. This deficiency can be compensated by using the appropriate binder. In this article, we present the influence of a water-soluble binder carrageenan on the electrochemical properties of the Li-S battery. The electrode with a carrageenan binder provides good stability during cycling and at high C-rates. Electrochemical testing was also carried out with a small prototype pouch cell with a capacity of 16 mAh. This prototype pouch cell with the water-based carrageenan binder showed lower self-discharge and low capacity drop. Capacity decreased by 7% after 70 cycles.

## 1. Introduction

The pressure to develop new batteries with higher gravimetric energy density is rising. There is an increasing demand for energy storage in modern applications, such as wearable electronics, electromobility, or stationary energy storage systems for renewable energy sources (photovoltaic or wind power plants, which are essential to meet climate goals). A major shift occurred with the launch of lithium-ion batteries (LiBs) on the market. LiBs offered much higher gravimetric energy densities compared to Ni-Cd and Ni-MH batteries available in the past [1]. However, the LiB technology, which is currently at the level of ~260 Wh kg^−1^ per cell, has its limits and may not cover the needs of new applications in the future despite its development [2]. One of the promising technologies that could meet even higher requirements is the technology of Li-S batteries. Their main advantage is the high theoretical capacity of sulfur (1675 mAh g^−1^), which is significantly higher than the theoretical capacity of LiFePO_4_ (170 mAh g^−1^) or LiNi_1/3_Mn_1/3_Co_1/3_O_2_ (280 mAh g^−1^). The disadvantage of Li-S batteries is the lower operational voltage against lithium ~2.1 V [3]. Despite this lower operational potential against lithium, the theoretical gravimetric energy density of sulfur is above 3000 Wh kg^−1^ [4]. In a practical application, Li-S batteries could exceed 500 Wh kg^−1^ [5]. Another advantage is the use of sulfur itself because it is abundant, cheap, and ecological compared to the currently used cobalt-based metal oxide cathode materials. Despite several advantages of Li-S batteries, their commercialization is obstructed by several major issues such as the insulating character of sulfur, high volumetric expansion of sulfur during cycling (~80%), and the polysulfide shuttle effect [6]. The polysulfide shuttle effect occurs during the charging and discharging of the Li-S battery. In the initial state, cyclo-S8 is reduced during discharging to higher lithium polysulfides (Li_2_S_8_, Li_2_S_6_, Li_2_S_4_) between 2.4 and 2.1 V vs. Li and then, between 2.1 and 1.7 V vs. Li, lower lithium polysulfides (Li_2_S_2_, Li_2_S) are formed. The lower lithium polysulfides are solid; however, higher lithium polysulfides are soluble in the electrolyte, which leads to the dissolution of the active material from the positive electrode. Soluble lithium polysulfides subsequently deposit on the lithium anode, recombine to lower lithium polysulfides, and increase the internal resistance of the cell. This shuttle effect, in combination with the volumetric expansion of sulfur, leads to high-capacity loss and low cycle life of the Li-S battery [7].

Currently, several methods to prevent the shuttle effect and decrease the influence of sulfur volumetric expansion are developed. A widely used technique is polysulfide trapping by porous carbon materials, which can bind lithium polysulfides in its porous structure and subsequently increase the conductivity of the electrode structure [8,9]. However, porous carbon materials cannot prevent polysulfide dissolution since their non-polar features are less efficient than polysulfide trapping [10]. Another technique is the preparation of interlayers, electrodes, or separator surface coatings which prevent lithium polysulfide shuttle and keep lithium polysulfides on the cathode side [11,12]. It has also been found that polar materials containing electronegative atoms such as N or O on their surface can electrochemically trap lithium polysulfides on their surface more efficiently compared to simple trapping in the porous carbon structure [13]. This is the reason N-doped carbon, polymeric carbon nitride (p-C_3_N_4_), WO_3_, Ti_4_O_7_, and MnO_2_ show catalytic effects in promoting the conversion of soluble lithium polysulfides, but also a strong electrostatic affinity between the catalyst and lithium polysulfides, which results in the long-term stability and fast electron transport during cycling [10]. Metal oxides can be, under some circumstances, used as additional active materials in the electrode with the use of cationic redox and anionic redox [14]. Another method to improve the cycling performance of Li-S batteries is by using a proper composition of the electrolyte. Additives such as LiNO_3_ or P_2_S_5_ help during the formation of a stable SEI layer on the Li surface, which improves cycle life and coulombic efficiency. On the other hand, additives such as manganese (II) acetylacetonate or ammonium salt NH_4_NO_3_ can be used to improve active sulfur utilization [15]. In addition to conventional liquid electrolytes, it is also possible to use solid electrolytes such as Li_6.4_La_3_Zr_1.4_Ta_0.6_O_12_ (LLZTO) or Li1 + xAlyGe_2_ − y(PO_4_)_3_ (LAGP) that prevent the polysulfide shuttle effect [16]. The sulfur structure itself can be modified by using sulfur-containing polymer prepared by vulcanization/inverse vulcanization methods. Cycle life or high C-rate performance can be improved thanks to the high versatility of polymer structures [17].

Another way to improve the properties of the Li-S battery is the use of a proper electrode binder. Binders soluble in organic solvents are commonly used. A typical representative of this group is poly(vinylidene) difluoride (PVDF). However, PVDF does not cope with mechanical stress during electrode cycling and has low swell ability in ether-based electrolytes, which leads to capacity losses during cycling [18]. Based on these findings, it is evident that the complexity of Li-S cathode behavior places greater requirements on the binder. This was the reason the investigation of other alternative binders began. It was observed that some binders containing oxygen, nitrogen, and halogen atoms could trap polysulfides and improve electrode performance [19]. In the case of poly(ethylene) oxide (PEO), a positive influence on the porosity of the electrode and polysulfide affinity was observed [20,21]. Strong polysulfide affinity was also observed in the case of polyvinylpyrrolidone PVP binder or in the case of poly(vinyl alcohol) (PVA) binder, which also decreases transport resistance of Li^+^ [22]. Another group of binders is the group of water-soluble binders. Water-soluble binders are more environmentally friendly, cheaper, and more interesting alternatives from the point of view of future applications. Another advantage of the water-soluble binders is that they are more hydrophilic and usually have free polar groups, which enhance polysulfide affinity [19]. A typical representative is sodium carboxymethylcellulose (NaCMC) which can form conductive pathways in electrode structure [23].

In this article, we propose the use of an organic binder carrageenan. Carrageenan is an available organic material extracted from seaweed. It is widely used in the medical and food industries. Carrageenan has several advantages: it is soluble in water, non-toxic, biodegradable, and can be synthesized with different concentrations of sulfate groups and different structures (kappa, iota, lambda) [24]. The presence of sulfate groups can improve the stability of Li-S batteries by polysulfide trapping, and the presence of a hydroxyl group can cause good adhesion of materials in the electrode structure [25]. We present tests of two carrageenan structures, iota and lambda, in this article. Lambda-carrageenan contains three sulfate groups per two galactose molecules. Iota-carrageenan contains two sulfate groups per two galactose molecules. It creates soft gels and has viscoelasticity properties. Iota-carrageenan was also reported in several publications as a possible biopolymer electrolyte [26]. It was observed that iota carrageenan has better performance at high C-rates and higher stability during cycling compared to lambda carrageenan and a standard PVDF binder. This is caused by the higher structural stability of the electrode, which leads to more stable resistance during cycling.

## 2. Experimental Section

### 2.1. Electrode Preparation and Cell Assembly

A basic electrode material slurry was prepared by stirring 60 wt% of sulfur (Sigma-Aldrich, St. Louis, USA), 30 wt% of Super P carbon (Timcal, Bodio, Switzerland), and 10 wt% of PVDF (polyvinylidene fluoride) (Sigma-Aldrich) binder in NMP (in N-methyl-2-pyrrolidone) (Sigma-Aldrich) solvent. The carrageenan-based electrode was prepared by stirring 60 wt% of sulfur (Sigma-Aldrich), 30 wt% of Super P carbon (Timcal), and 10 wt% of iota carrageenan or lambda carrageenan (CPKelco, Nijmegen, Netherlands) binder in water. After 24 h of mixing, all electrode slurries were deposited on an aluminum foil by a coating bar and subsequently dried in the oven at 60 °C for 24 h and pressed with the pressure of 350 kg cm^−2^. Disk electrodes with a diameter of 18 mm were subsequently cut out of the coated aluminum foils. The basic PVDF-based electrode will be marked as S-PVDF and the iota carrageenan and lambda carrageenan electrodes as S-Car-Iota and S-Car-Lamb, respectively. All three types of electrodes (S-PVDF, S-Car-Iota, S-Car-Lamb) were dried again in a vacuum and then in an oven at 60 °C inside a glove box (Jacomex, Dagneux, France). The sulfur loading of all electrodes was ~1.5 mg cm^−2^. The assembly of the test cells ECC-STD (El-Cell^®^, Hamburg, Germany) was carried out in a glove box in an argon atmosphere. A metal lithium disc was used as a counter electrode, and 0.25 mol L^−1^ solution of lithium nitrate (LiNO_3_) (Sigma-Aldrich) + 0.7 mol L^−1^ of lithium bis(tri-fluoromethanesulfonyl)imide (LiTFSI) (Sigma-Aldrich) in 1,2-dimethoxyethane (DME) (Sigma-Aldrich) and 1,3-dioxolane (DOL) (Sigma-Aldrich) was used as electrolyte with the volume ratio of 2:1. The electrolyte was impregnated into a glass fiber-based separator.

The electrode for the pouch cell, prepared at Dipro University with the size of 7 cm × 3 cm, was coated by a slurry prepared by stirring of 60 wt% of sulfur (Sigma-Aldrich), 9 wt% of Super P carbon (Timcal), 21% of KJ black carbon (Lion Specialty Chemicals, Tokyo, Japan), and 10 wt% of PVDF (Sigma-Aldrich) binder in NMP (Sigma-Aldrich) solvent in the case of the S-PVDF electrode. The S-Car-Iota and S-Car-Lamb pouch cell electrodes were coated by a slurry prepared by stirring of 60 wt% of sulfur (Sigma-Aldrich), 9 wt% of Super P carbon (Timcal), 21% of KJ black carbon (Lion Specialty Chemicals), and 10 wt% of the respective carrageenan (CPKelco) in water. The pouch cell electrodes were dried in an oven at 60 °C for 24 h and pressed with a pressure of 350 kg cm^−2^. The electrodes were then additionally dried in a vacuum oven at 60 °C. The pouch cells were subsequently assembled in the glow box with an argon atmosphere. A metal lithium foil was used as a counter electrode, and 0.25 mol L^−1^ LiNO_3_ + 0.7 mol L^−1^ LiTFSI in DME:DOL 2:1 *v*/*v* electrolyte was impregnated into a Celgard 3500 separator (Celgard LLC, Charlotte, USA). The sulfur loading of the S-Car-Iota pouch cell electrode was 1.42 mg cm^−2^, and it was 1.26 mg cm^−2^ for the S-PVDF pouch cell electrode.

### 2.2. Characterization Methods

All electrochemical measurements were performed using a VMP3 potentiostat (Bio-Logic). Cyclic voltammetry (CV) and galvanostatic cycling were used for electrochemical characterization of electrodes set up in a test cell ECC-STD (El-Cell^©^). Both methods were performed on a VMP3 potentiostat (Bio-Logic, Seyssinet-Pariset, France). CV was performed in the potential window from 1.8 to 3.0 V vs. Li/Li^+^. The scan rate was set to 0.1 mV s^−1^. Galvanostatic cycling was carried out within a potential window from 1.8 to 2.8 V vs. Li/Li^+^. The capacities obtained at different C-rates from 0.2 C to 1 C were related to the weight of sulfur. Electrochemical impedance spectra (EIS) data were measured within the frequency range from 0.01 Hz to 1 MHz with the alternating current voltage amplitude of 5 mV. Scanning electron microscopy (SEM) and energy-dispersive X-ray spectroscopy (EDS) of the electrode structure was performed on a TESCAN VEGA3 XMU electron microscope with a Bruker EDAX analyzer (Bruker, Billerica, USA).

## 3. Results and Discussion

**Figure 1:**
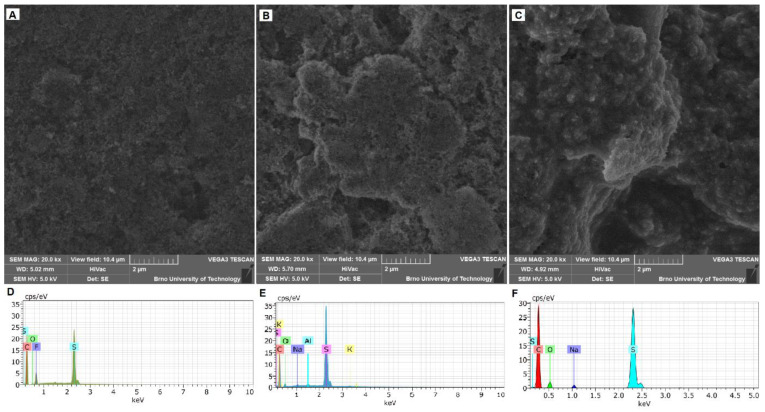
SEM images (field of view 10.4 µm) and EDS spectra of the electrode surface: (**A**) S-PVDF electrode surface, (**B**) S-Car-Iota electrode surface, (**C**) S-Car-Lamb electrode surface (**D**) S-PVDF EDS spectra, (**E**) S-Car-Iota EDS spectra, (**F**) S-Car-Lamb EDS spectra.

We can see the surface structures of the S-PVDF, S-Car-Iota, and S-Car-Lamb electrodes investigated by SEM and EDS in Figure 1. The used field of view for all electrodes is 20.8 µm. The surface structure of the S-PVDF electrode is shown in Figure 1A. The used field of view is the same as for the other SEM pictures—10.4 μm. As we can see, the electrode is highly porous, and the individual particles are well distinguishable. SEM pictures of the S-Car-Iota electrode surface can be seen in Figure 1B). The surface is still very porous, and individual particles are more connected compared to the S-PVDF electrode. The surface of the S-Car-Lamb electrode is uniform and less porous compared to the other electrodes. Individual particles are completely covered with the binder, see Figure 1C. The EDS spectra of electrodes are shown in Figure 1D (S-PVDF electrode), Figure 1E (S-Car-Iota electrode), and Figure 1F (S-Car-Lamb electrode). All electrodes contain sulfur, carbon, and oxygen. The presence of fluorine from the PVDF binder is also evident in the case of the S-PVDF electrode. In the case of the S-Car-Lamb and S-Car-Iota electrodes, we can see the presence of sodium and potassium contained in the binder, respectively.

**Figure 2:**
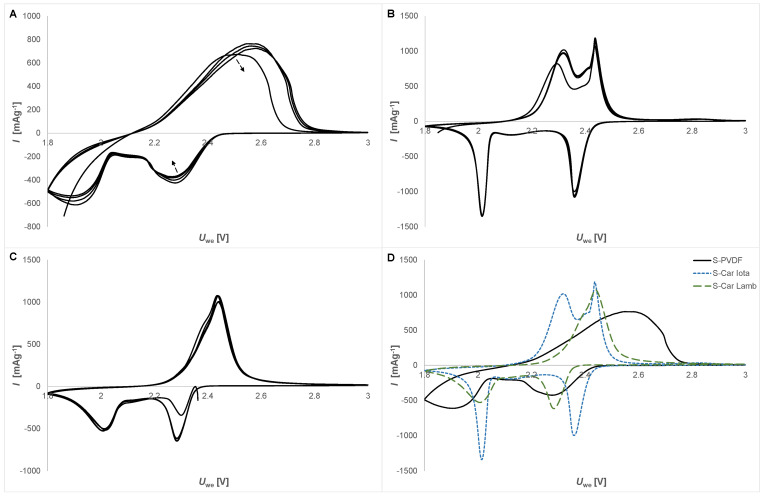
The CV profiles obtained at the scan rate of 0.1 mV s^−1^: (**A**) S-PVDF electrode, (**B**) S-Car-Iota electrode, (**C**) S-Car-Lamb electrode, (**D**) and comparison of the second CV cycle of all electrodes.

CV analysis was used to study the redox reactions and kinetics of the studied electrodes. The results of CV analysis are shown in Figure 2A (S-PVDF electrode), Figure 2B, (S-Car-Iota electrode), and Figure 2C (S-Car-Lamb electrode), and the comparison of the second cycle of CV is in Figure 2D. The kinetics of the S-PVDF electrode is lower compared to the other investigated electrodes. The anodic and cathodic peaks connected with the red/ox reactions of higher and lower lithium polysulfides are not sharp, and electrochemical activity decreases with cycle number. We can see nice sharp anodic peaks at 2.32 and 2.43 V and two cathodic peaks at 2.36 and 2.0 V related to higher and lower lithium polysulfides, respectively, in the case of the S-Car-Iota electrode. The S-Car-Lamb electrode also shows sharp anodic peaks; however, the anodic peaks are closer at 2.4 and 2.44 V, the higher cathodic peak is at lower potential (2.28 V), and the lower cathodic peak is at 2.0 V.

**Figure 3:**
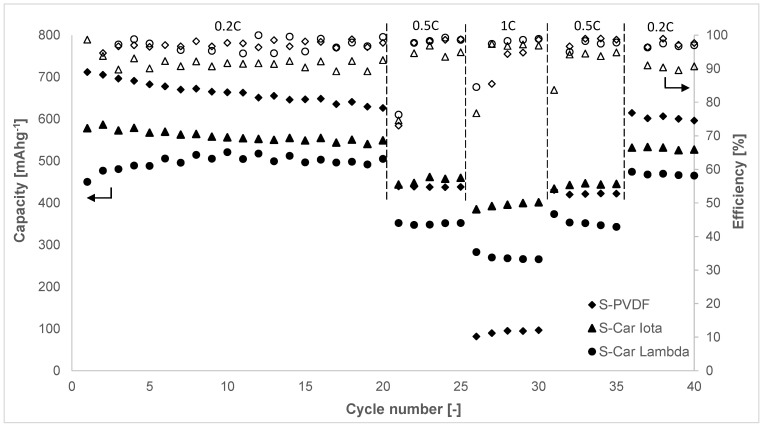
Galvanostatic cycling of the S-PVDF, S-Car-Iota, and S-Car-Lamb electrodes at different C-rates.

Figure 3 shows results from galvanostatic cycling of the studied electrodes at different C-rates. The S-PVDF electrode exhibits higher capacity at the beginning of cycling (712 mAh g^−1^) compared to the S-Car-Iota (578 mAh g^−1^) and S-Car-Lamb (450 mAh g^−1^) electrodes. However, the stability of the S-PVDF electrode is worse, and the capacity drop at 1 C rate is 86% while the S-Car-Iota and S-Car-Lamb electrodes showed capacity drops of 32% and 44%, respectively. The capacity drop between cycle 20 at 0.2 C and the last cycle of the whole cycling was 5% for the S-PVDF electrode, 4% for the S-Car-Iota electrode, and 8% for the S-Car-Lamb electrode. The overall capacity drop of the S-PVDF electrode was 16%, while it was 8% for the S-Car-Iota electrode. The S-Car-Lamb electrode showed a slight capacity increase (about 3%) because of the electrode formation at the beginning of cycling. The S-Car-Iota electrode exhibits lower efficiency at the C-rate of 0.2 C. The efficiency at the high rate of 1 C is similar or higher compared to other electrodes. This can be optimized in the future by modification of pressure during pressing, which will improve the utilization of the electrode. The carrageenan-based binder electrodes exhibit higher stability during cycling. The S-Car-Iota electrode also shows better stability at higher C-rates and higher capacity compared to the S-Car-Lamb electrode. This is because of the better porosity of the electrode, which was observed by SEM analysis. The S-Car-Lamb electrode was completely glued by the binder, which decreased the surface area and capacity of the electrode. The highest porosity was observed by SEM in the case of the S-PVDF electrode, which explained the higher capacity during low C-rates caused by higher surface area. However, contact between particles and structural stability was worse, leading to a higher capacity drop at higher C-rates and during cycling. Gao et al. [27] reported capacity drops of ~41%, ~39%, and 49% after 50 cycles at the current density of 122 mA g^−1^ for electrodes with polytetrafluoroethylene (PTFE), acrylonitrile copolymer LA132, and polyaniline (PANI) binder (~53% S content in the electrode and the loading of 1.0–1.4 mg cm^–2^). Cheng et al. [28] reported the capacity drop of 53% for an electrode with PVDF and 25% of the sulfonated polystyrene (SPS) binder after 100 cycles at 200 mA g^−1^ (52% S content in the electrode and the loading of ~1.5 mg cm^–2^). Hernández et al. [29] reported the capacity drop of 70% after 30 cycles at 0.2 C for the electrode with poly(ethylene oxide) (PEO) and naphthalene polyimide-PEO binder (~60% S content in the electrode and the loading of 0.8–1.0 mg cm^–2^). The capacity drop of 58% after 100 cycles at 0.2 C for the PVDF binder-based electrode and 27% for the poly(vinylidene difluoride-trifluoroethylene) (P(VDF-TRFE)) binder-based electrode (49% S content in the electrode) was reported by Wang [30]. In the case of a water-soluble binder, Godoi et al. [31] reported the capacity drop of 48% for the electrode based on sodium alginate (NaAlg) after 100 cycles at 0.2 C (40% S content in the electrode). Lu et al. [32] demonstrated the capacity drop of 26% after 150 cycles at 0.2 C with guar gum (GG) binder-based electrode (~51% S content in the electrode and the loading of 0.6–0.7 mg cm^–2^). Yang et al. [33] reported the use of a polyelectrolyte water-soluble binder. He reported better kinetics compared to the PVDF binder; the overall capacity drop after 50 cycles was ~25%, and the capacity drop at 1 C was ~39% during cycling at different C-rates from 0.2 to 1 C (50.4% S content in the electrode and the loading of 1 mg cm^–2^). Chen et al. [34] reported the capacity drop of ~37% after 50 cycles at different C-rates from 0.2 C to 2 C in the case of a chitosan binder-based electrode (~63% S content in the electrode and the loading of 1.0–1.5 mg cm^–2^).

**Figure 4:**
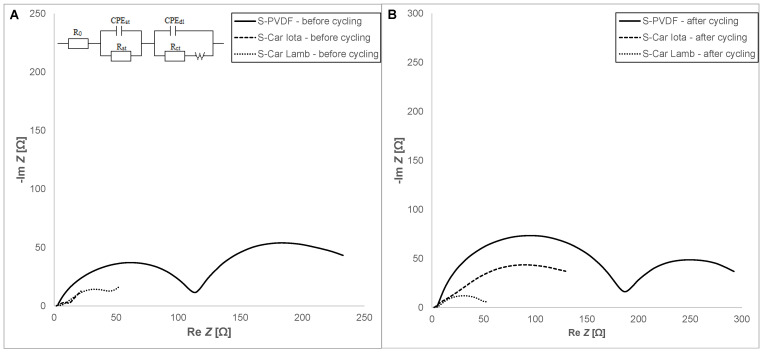
The electrochemical impedance spectra for the S-PVDF, S-Car-Iota, and S-Car-Lamb electrodes: (**A**) before cycling, (**B**) after cycling.

The Nyquist plots of the S-PVDF, S-Car-Iota, and S-Car-Lamb electrodes before cycling are shown in Figure 4A and the plots after cycling are in Figure 4B. Equivalent circuits used for fitting are according to Zu et al. [35]. Based on the analysis of impedance, the highest charge transfer resistance R_ct_ before cycling was observed in the case of the S-PVDF electrode (112 Ω). The S-Car-Iota and S-Car-Lamb showed lower R_ct_ of 34 and 79 Ω, respectively. The R_ct_ of the S-PVDF after cycling was significantly higher (215 Ω). On the other hand, R_ct_ of the S-Car-Iota electrode decreased to 24 Ω. These decreases of R_ct_ can be connected with the SEI formation with better stability and better interfacial reactions. Low Rct is in correlation with the results obtained by CV and galvanostatic cycling, where S-Car-Iota showed the best reaction kinetics. Similar behavior was also observed in the case of the S-Car-Lamb electrode, where R_ct_ decreased to 56 Ω.

**Figure 5:**
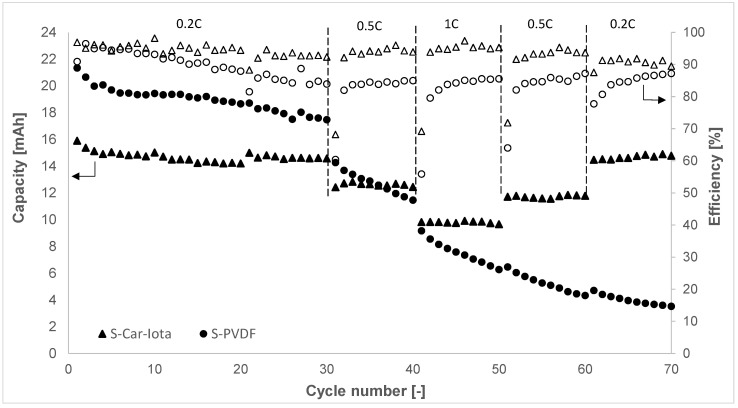
Galvanostatic cycling of the S-PVDF and S-Car-Iota pouch cells.

According to the previous results, it was decided to set up two pouch cells with an electrode size of 7 cm × 3 cm, based on the S-PVDF and S-Car-Iota electrodes. These pouch cells were cycled at different C-rates from 0.2 to 1 C for 70 cycles (Figure 5). The S-PVDF pouch cell exhibited higher capacity at the beginning of the cycling (21.3 mAh; 804 mAh g^−1^) compared to the S-Car-Iota pouch cell (15.9 mAh; 532 mAh g^−1^). However, the stability of the S-PVDF pouch cell was much worse. The capacity drop of the S-PVDF pouch cell during the first 30 cycles at 0.2 C was about 18%, and it was 8% for the S-Car-Iota pouch cell. The capacity of the S-PVDF pouch cell decreased even more significantly during the subsequent cycling at higher C-rates. The overall capacity drop of the S-PVDF pouch cell was 83%. The S-Car-Iota pouch cell was more stable with a capacity drop of 39% at a 1 C rate compared to the first cycle at 0.2 C, and the overall capacity drop was 7%. These results are very similar to the results obtained in the electrochemical test cell. Lecey et al. [21] reported a capacity drop ~50% after 50 cycles with a pouch cell with an electrode size of 3 cm × 3 cm based on a PEO binder (50% S content in the electrode and the loading of 0.3–0.6 mg cm^–2^). Salihoglu et al. [36] reported an ~85% capacity drop after 100 cycles for a 3 Ah cell. Huang et al. [37] reported the capacity drop of 12% after 30 cycles at 0.05 C for a 1.5 Ah cell with an S-KJ black composite-based electrode in combination with a LA132 aqueous binder and carbon-coated electrolyte (63% S content in the electrode and the loading of 3.0 mg cm^–2^).

**Figure 6:**
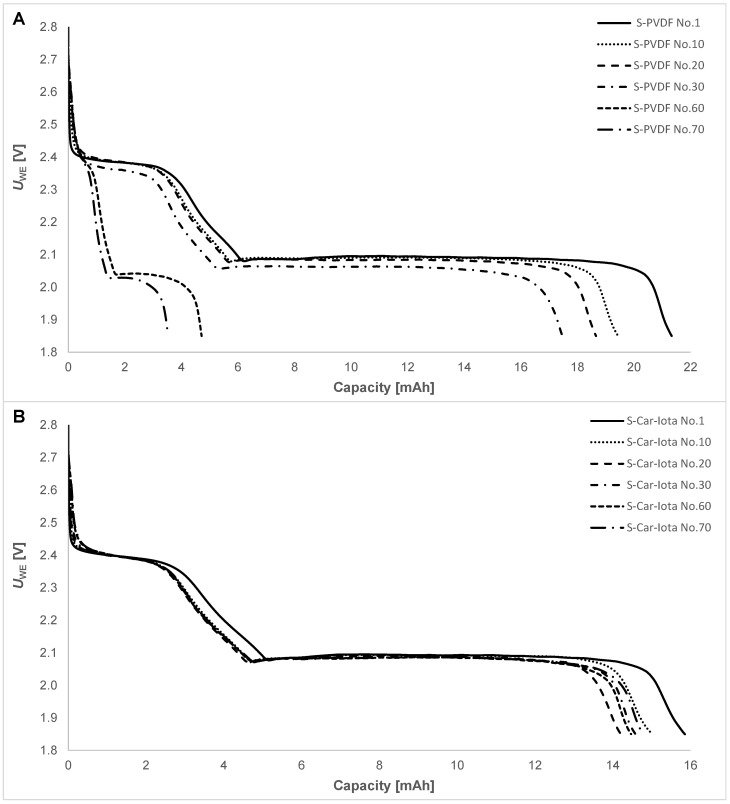
Discharge curves of the (**A**) S-PVDF and (**B**) S-Car-Iota pouch cells during different cycles.

A comparison of the discharge cycles of the S-PVDF and S-Car-Iota pouch cells is shown in Figure 6. It is evident that, during cycling, S-PVDF (Figure 6A) is losing the potential of the higher and lower discharge plateaus and the higher discharge plateau related to polysulfide shuttle decreases in connection with the active material losses.

However, the S-Car-Iota pouch cell is very stable. The potentials of the higher and lower discharge plateaus stay the same, and capacity at the higher discharge plateau remains the same during the whole cycling. The electrode with the carrageenan iota binder can prevent polysulfide shuttle and compensate for the volumetric expansion of sulfur during cycling.

## 4. Conclusions

In summary, we have prepared Li-S cells based on environmentally friendly water-soluble binders based on carrageenan. The use of an organic binder can decrease the price of Li-S batteries, decrease their production costs, and increase the environmental friendliness of this battery technology in the future. The results prove that the use of carrageenan iota as a binder has a positive influence on the stability of the electrode at higher rates and during the whole cycling. The S-Car-Iota electrode exhibits the lowest charge transfer resistance before and after cycling and better kinetics. The capacity of the S-Car-Iota electrode (578 mAh g^−1^) compared to the standard PVDF electrode (712 mAh g^−1^) was lower, probably because of lower porosity due to higher binding properties. The results from a small electrochemical cell were also proven by measurements of a pouch cell battery. The S-Car-Iota pouch cell exhibits high stability with a low capacity drop of 7% after 70 cycles. The capacity drop at the C-rate of 1 C was 37% in the case of the S-Car-Iota pouch cell. A similar performance was also observed during cycling in the electrochemical test cell, where the capacity drop of the S-Car-Iota electrode was 32%. This was due to the ability of the carrageenan iota binder to retain polysulfides more efficiently in the electrode structure. The binding properties of the carrageenan binder were for both electrodes very high. Future research could be focused on the decrease in binder mass in the electrodes and the increase in the active sulfur ratio, which increases the energy density of the electrode. Another option could be optimization of active mass preparation by pre-milling of a carrageenan binder with sulfur and carbon conductive additives.

## Data Availability

The data presented in this study are available on request from the corresponding author.
